# Hematological markers and ultrasound 7-joint inflammation score as add-on tools in the assessment of inflammation in rheumatoid arthritis patients

**DOI:** 10.1186/s43166-022-00126-0

**Published:** 2022-05-04

**Authors:** Abd Elatif Ahmed Gaballah, Noha Abdelhalim Elsawy, Wessam Mohamed El Gendy, Ahmed Hafez Afifi, Maha Saeid Mahmoud Hanafi

**Affiliations:** 1 Department of Physical Medicine, Rheumatology and Rehabilitation, Faculty of Medicine, Alexandria University, Alexandria, Egypt; 2 Department of Clinical and Chemical pathology, Faculty of Medicine, Alexandria University, Alexandria, Egypt; 3 Department of Radiodiagnosis, Faculty of Medicine, Alexandria University, Alexandria, Egypt

**Keywords:** Hematological markers, Ultrasound 7-joint inflammation score, DAS28 CRP, DAS28 ESR

## Abstract

**Background:**

Rheumatoid arthritis (RA) is an autoimmune disease characterized by synovial inflammation and joint destruction that eventually induces severe disability. Inflammation is the key determinant and primary underlying mechanism leading to disability and increased mortality in patients with RA. This study aimed to correlate the hematological markers and ultrasound 7-joint inflammation score to disease activity in rheumatoid arthritis patients.

**Results:**

The current study involved 54 RA patients diagnosed according to the 2010 ACR/EULAR classification criteria for RA and thirty healthy control subjects. There were 48 females (88.9%) and 6 males (11.1%). The age of patients ranged from 32 to 60 years, with a mean of 46.04 ± 5.65 years. Using disease activity score 28-ESR, total white blood cell count and absolute lymphocytic count were significantly lower in RA patients with high disease activity. Neutrophil-to-lymphocyte ratio, platelet-to-lymphocyte ratio, mean platelet volume, ESR, and CRP were significantly higher in patients with high disease activity using disease activity score 28 CRP. Also, a statistically significant positive correlation was detected between neutrophil-to-lymphocyte ratio and platelet-to-lymphocyte ratio and both clinical disease activity scores. Red cell distribution width but not platelet distribution width was significantly higher in RA patients but both parameters had no association or correlation with clinical disease activity scores. Neutrophil-to-lymphocyte ratio was found to have a statistically significant positive correlation with the tenosynovitis score by powered Doppler ultrasound. There were statistically significant positive correlations between disease activity score 28 ESR and CRP and synovitis and tenosynovitis scores by greyscale and powered Doppler ultrasound using the ultrasound 7 score.

**Conclusion:**

Neutrophil-to-lymphocyte ratio, platelet-to-lymphocyte ratio, and mean platelet volume could be potential inflammatory markers for follow-up of disease activity in RA patients. The ultrasound 7 score is a simple and practical scoring system for use in the detection of inflammation, even subclinically in RA patients, which may help the physician in his clinical decisions. The combined use of both hematological markers and the ultrasound 7 score may be of great value.

## Background

Rheumatoid arthritis (RA) is an autoimmune disease characterized by synovial inflammation and joint destruction that eventually induces severe disability [1]. Inflammation is the key determinant and primary underlying mechanism leading to disability and increased mortality in patients with RA [2].

The pathogenesis of RA is a complex process in which there is an interaction between genetic and environmental factors, resulting in the activation of the innate and adaptative immune systems that result in autoantigen presentation with antigen-specific T and B cell activation and inflammatory cytokine production. This cascade of events leads to synovitis, synovial proliferation, cartilage, and subchondral bone destruction [3].

C-reactive protein (CRP) and erythrocyte sedimentation rate (ESR) are the most widely used assays for the assessment of inflammation in RA due to their reliability, reproducibility, and cost-effectiveness. On the other hand, ESR is influenced by several factors unrelated to inflammation, such as age, sex, anemia, and renal failure. Rapid serum level change in response to inflammation is one of the major advantages of CRP compared to ESR, although it shares some of the disadvantages of ESR [4].

Systemic inflammation is associated with alterations in circulating blood cells’ quantity and composition. Indeed, normochromic anemia, thrombocytosis, neutrophilia, and lymphopenia usually accompany many inflammatory conditions. Therefore, the features of circulating blood cell components can be used for the assessment of inflammatory activity [5].

Neutrophil-to-lymphocyte ratio (NLR) and platelet-to-lymphocyte ratio (PLR) have been investigated as inflammatory markers for the assessment of inflammation in many inflammatory, cardiovascular, and malignant diseases [6, 7].

Platelets play an important role in the pathophysiology of RA through their contributions to inflammation and immune modulation. Activated platelets release pro-inflammatory micro-particles that interact with WBCs, leading to joint and systemic inflammation [8–10]. Platelet count, mean platelet volume (MPV), and platelet distribution width (PDW) are three useful indices of platelet function that can be easily measured in routine complete blood counts and are thought to be mirror images of platelet function [8, 11].

Red blood cell distribution width (RDW) is a parameter that reflects the heterogeneity of erythrocyte volume. It is mainly used to differentiate types of anemia, but it is correlated with the level of inflammation in many diseases, such as inflammatory bowel disease, systemic lupus erythematosus, or Behçet’s disease [12].

These markers are inexpensive and easily obtained. However, their serum levels in RA patients compared to healthy subjects as well as their relation to RA disease activity showed controversial results [13–15].

Musculoskeletal ultrasound has been widely used in rheumatological practice. Gray-scale (GS) musculoskeletal ultrasound usually identifies synovial proliferation, but power Doppler (PD) is capable of recognizing active inflammation and neoangiogenesis. Both parameters are very useful in the follow-up of RA patients. In addition, it is also reliable for the detection of bone erosions, subclinical synovitis, and prediction of disease relapse and structural progression [16, 17].

Ultrasound has many advantages, such as its relatively low cost, wide availability, lack of contraindications, and non-invasive real-time imaging capabilities. But, in spite of these very important advantages, ultrasound is considered an operator-dependent technology, which is considered a disadvantage [18]. The ultrasound 7 score is a semiquantitative US scoring system that has been proposed to assess established RA and other inflammatory arthropathies [19].

The aim of the present study was to correlate the hematological markers and ultrasound 7-joint inflammation score with disease activity in rheumatoid arthritis patients.

## Methods

Patients in the current study were recruited from those attending the Out-Patient Clinics of Physical Medicine, Rheumatology, and Rehabilitation Department. In total, our case-control observational study included 54 RA patients fulfilling the 2010 ACR/EULAR classification criteria for RA [20], and thirty healthy control subjects with matching age and sex were enrolled in the study. Patients with any inflammatory arthropathies, cancer, acute or chronic infection, chronic renal failure, and those who were pregnant or in the recent post-partum period (6 months) were excluded from the study. Disease activity was assessed using disease activity score (DAS) 28-ESR [21] and DAS28-CRP [22]. Both disease activity 28 scores were used to investigate which one is more correlated with the inflammatory markers and the US7 score was investigated in the current study. The history of the current medications received by patients was recorded with no modifications done on the treatment plan.

Venous blood was collected from each patient into tubes with and without anticoagulants. Standard laboratory workup included: complete blood count (CBC) [23] using Sysmex- XT1800i apparatus. From which the following data were collected: hemoglobin level; red blood cells count (RBCs); white blood cells count (WBCs); platelets count; absolute neutrophils and lymphocytes count; red cell distribution width (RDW) and markers of platelet activation (MPV and PDW). NLR was calculated by dividing the absolute count of neutrophils by the absolute count of lymphocytes [24]; PLR was calculated by dividing the absolute platelet count by the absolute lymphocytic count [24]. ESR was measured immediately after blood collection using the Westergren method [25]. Rheumatoid factor [26] and CRP levels [27] were measured by nephelometric methods.

Patients were examined using a Toshiba Xario 200 ultrasound system by the same sonographer. The Gray Scale (GS) and Power Doppler (PD) examinations were done using a high-frequency broadband linear array transducer, which was used at 10-18 MHz. The US examination was performed in two perpendicular planes, according to EULAR guidelines [28]. We followed the OMERACT standardized definitions of US pathological findings [29]. The joints to be examined included: the wrist, the 2^nd^ metacarpophalangeal joint (MCP2), the 3rd metacarpophalangeal joint (MCP3), the 2^nd^ proximal interphalangeal joint (PIP2), the 3rd proximal interphalangeal joint (PIP3), the 2^nd^ metatarsophalangeal joint (MTP2) and the 5th metatarsophalangeal joint (MTP5). The tendons to be examined included: all tendons in the flexor and extensor compartments of wrist, long flexors and extensors of fingers. The ultrasonographic examination was done bilaterally but the score was calculated using the parameters of the most symptomatizing side.

The examined joints were scored according to the US7 score including the sum of synovitis scores in the GSUS (0–27) and PDUS (0–39) modes, tenosynovitis/paratenonitis in the GSUS (0–7) and PDUS (0–21) modes, and erosions (0–14) in the GSUS mode [30]. The US remission was defined as GS ≤ 1 and PD = 0 [31, 32].

## Statistical analysis

Data were fed to the computer and analyzed using IBM SPSS software package version 20.0. Qualitative data were described using numbers and percentages while range mean, standard deviation, median, and interquartile range (IQR) were used for quantitative data. The Kolmogorov-Smirnov test was used to verify the normality of distribution. The significance was judged at the 5% level. The Chi-square test was used to compare between different groups. Fisher’s exact or Monte Carlo correction were used for correction for chi-square when more than 20% of the cells have an expected count less than 5. Student *t* test was used for comparing normally distributed quantitative variables between groups. The Mann-Whitney test was used for abnormally distributed quantitative variables while the Pearson coefficient was used to for normally distributed quantitative variables. The Spearman coefficient was used to correlate between two abnormally distributed quantitative variables.

## Results

There were 48 females (88.9%) and 6 males (11.1%). The age of patients ranged from 32 to 60 years, with a mean of 46.04 ± 5.65 years. The disease duration of the studied patients ranged from 1 to 22 years, with a mean of 8.74 ± 6.14 years. As regards the DAS28-ESR score, 9 patients (16.7%) were in clinical remission, 8 patients (14.8%) had low disease activity (LDA), 22 patients (40.7%) had moderate disease activity (MDA) and 15 patients (27.8%) had high disease activity (HDA), the score ranged from 1.90 to 7.95, with a mean of 4.32 ± 1.54. As regards the DAS28-CRP score, 8 patients (14.8%) were in clinical remission, 10 patients (18.5%) were in LDA, 21 patients (38.9%) were in MDA, and 15 patients (27.8%) were in HAD. The score ranged from 1.13 to 6.81, with a mean of 3.71 ± 1.36.

There was a statistically significant difference between the two groups as regards hemoglobin level, RBCs count, the absolute neutrophilic count which were lower in RA patients, and RDW which was higher in RA patients. Anemia was diagnosed in 55.6% of RA patients. There was no statistically significant difference between the two groups as regards platelet count, total WBCs count, the absolute lymphocytic count, NLR, PLR, MPV, and PDW (Table 1).

**Table 1:** Comparison between the two studied groups according to blood cell count and inflammatory markers

	Patient (***n*** = 54)	Control (***n*** = 30)	***P***
**Hemoglobin (g/dl)**			
Min.–Max.	9.50–16.20	12.0–16.0	<0.001*
Mean ± SD.	12.24 ± 1.29	13.63 ± 1.21	
**Red blood cells( × 106/ul)**			
Min.–Max.	3.61–5.91	3.94–6.40	0.007*
Mean ± SD.	4.54 ± 0.52	4.89 ± 0.61	
**Platelets (× 103/ul)**			
Min.–Max.	116.0–410.0	201.0–462.0	0.081
Mean ± SD.	251.74 ± 74.28	281.23 ± 71.37	
**White blood cells (× 103/ul)**			
Min.–Max.	3.48–14.30	4.0–10.38	0.080
Mean ± SD.	6.11 ± 1.89	6.86 ± 1.74	
**Neutrophils (× 103/ul)**			
Min.–Max.	1.33–9.58	2.15–5.95	0.030*
Median (IQR)	3.16 (2.39–4.11)	3.83 (3.15–4.53)	
**Lymphocytes (× 103/ul)**			
Min.–Max.	0.80–4.05	1.04–3.43	0.103
Median (IQR)	1.92 (1.46–2.31)	2.11 (1.66–2.68)	
**PDW (fl)**			
Min.–Max.	10.50–16.70	10.10–15.70	0.277
Mean ± SD.	12.94 ± 1.57	12.57 ± 1.39	
**MPV (fl)**			
Min.–Max.	0.20–13.50	8.0–11.80	0.302
Median (IQR)	9.95 (9.0–11.0)	10.60 (9.70–10.80)	
**RDW (%)**			
Min.–Max.	10.20–18.0	12.20–15.50	0.008*
Mean ± SD.	14.16 ± 1.73	13.25 ± 0.69	
**NLR**			
Min.–Max.	0.58–4.95	0.58–3.39	0.978
Median (IQR)	1.70 (1.31–2.33)	1.71 (1.51–2.18)	
**PLR**			
Min.–Max.	58.72–338.87	69.39–212.50	0.823
Median (IQR)	135.89 (93.44–181.37)	118.35(103.61–172.62)	

*IQR* Interquartile range, *PDW* Platelet distribution width, *MPV* Mean platelet volume, *RDW* Red cell distribution width, *NLR* Neutrophil-lymphocyte ratio, *PLR* Platelet-lymphocyte ratio

*Statistically significant at *P*≤ 0.05, *P* value ≤0.01: highly significant, *t* Student’s *t* test, *U* Mann-Whitney test

There was a statistically significant association between disease activity categories of DAS 28 ESR and total WBCs count (*P* = 0.036), absolute lymphocytic count (*P* = 0.016), and mean platelet volume (*P* = 0.034) (Table 2). There was a statistically significant association between disease activity categories in DAS 28 CRP and absolute lymphocytic count (*P* = 0.022), neutrophil-to-lymphocyte ratio (*P* = 0.018) and platelet lymphocyte ratio (*P* = 0.027) (Table 3).

**Table 2:** Comparison between DAS 28 ESR categories regarding blood cell count and inflammatory markers

	DAS 28 (ESR)				***P***
	Remission (***n*** = 9)	LDA (***N*** = 8)	MDA (***N*** = 22)	HAD (***N*** = 15)	
**Hemoglobin (g/dl)**					
Min.–Max.	9.50–16.20	10.10–14.40	9.80–14.20	9.60–13.30	0.167
Mean ± SD.	13.09 ± 2.09	12.31 ± 1.32	12.04 ± 1.02	11.98 ± 0.90	
**RBCs( ×10**^**6**^**/μl)**					
Min.–Max.	3.81–5.84	4.17–5.91	3.61–5.21	3.74–5.21	0.198
Mean ± SD.	4.54 ± 0.61	4.90 ± 0.58	4.47 ± 0.50	4.45 ± 0.40	
**Platelets (×10**^**3**^**/μl)**					
Min.–Max.	127.0–347.0	171.0–410.0	116.0–397.0	116.0–322.0	0.907
Mean ± SD.	244.4 ± 64.12	266.0 ± 99.47	254.8 ± 75.91	244.1 ± 68.69	
**WBCs (×10**^**3**^**/μl)**					
Min.–Max.	3.48–8.38	6.66–8.93	3.48–14.30	4.07–7.83	0.036*
Mean ± SD.	5.54 ± 1.50	7.86 ± 0.92	5.92 ± 2.36	5.82 ± 1.16	
**Neutrophils (×10**^**3**^**/μl)**					
Min.–Max.	1.50–4.82	2.63–5.10	1.33–9.58	2.18–5.45	0.051
Median	2.19	4.16	3.0	3.17	
**Lymphocytes (×10**^**3**^**/μl)**					
Min.–Max.	0.96–2.72	1.73–3.40	0.87–4.05	0.80–2.40	0.016*
Median	1.93	2.74	1.87	1.72	
**ESR (mm/h)**					
Min.–Max.	8.0–30.0	5.0–35.0	10.0–60.0	10.0–72.0	<0.001*
Mean ± SD.	16.56 ± 8.78	11.20 ± 9.75	28.32 ± 13.96	45.0 ± 20.17	
**CRP (mg/dl)**					
Min.–Max.	0.60–6.40	1.20–12.90	0.64–23.0	0.64–20.70	0.031*
Mean ± SD.	3.10 ± 1.67	4.08 ± 3.75	7.82 ± 6.53	9.47 ± 6.04	
**PDW (fl)**					
Min.–Max.	11.60–14.30	11.50–15.10	10.50–16.70	11.0–14.90	0.749
Mean ± SD.	12.72 ± 0.97	13.21 ± 1.45	12.74 ± 2.01	13.23 ± 1.21	
**MPV (fl)**					
Min.–Max.	7.70–13.50	7.10–10.80	8.50–13.50	0.20–11.80	
Median	10.30	8.80	10.45	9.80	
**RDW (%)**					
Min.–Max.	12.40–16.80	12.50–18.0	11.10–18.0	10.20–16.90	
Median	13.10	14.70	13.75	14.10	
**NLR**					
Min.–Max.	0.59–2.49	1.02–2.66	0.58–4.95	1.30–3.70	
Median	1.32	1.65	1.68	1.86	
**PLR**					
Min.–Max.	73.80–198.2	59.11–145.9	58.72–338.8	72.70–338.9	0.134
Median	132.2	117.7	134.2	170.3	

*DAS* Disease activity score, *LDA* Low disease activity, *MDA* Moderate disease activity, *HDA* High disease activity, *RBCs* Red blood cells, *WBCs* White blood cells, *ESR* Erythrocyte sedimentation rate, *CRP* C reactive protein, *PDW* Platelet distribution width, *MPV* Mean platelet volume, *RDW* Red cell distribution width, *NLR* Neutrophil-lymphocyte ratio, *PLR* Platelet-lymphocyte ratio

*Statistically significant at *P*≤ 0.05, *P* value ≤0.01: highly significant

**Table 3:** Comparison between DAS 28 (CRP) categories regarding blood cell count and inflammatory markers

	DAS 28 (CRP)				***P***
	Remission (***n*** = 9)	LDA (***n*** = 8)	MDA (***n*** = 22)	HAD (***n*** = 15)	
**Hemoglobin (g/dl)**					
Min.–Max.	9.50–16.20	10.10–14.50	9.80–13.60	9.60–13.60	0.183
Mean ± SD.	12.84 ± 2.14	12.73 ± 1.48	12.02 ± 0.85	11.89 ± 1.01	
**RBCs (× 10**^**6**^**/μl)**					
Min.–Max.	3.81–5.84	3.89–5.91	3.61–5.21	3.74–5.21	0.407
Mean ± SD.	4.58 ± 0.67	4.78 ± 0.57	4.46 ± 0.49	4.46 ± 0.42	
**Platelets (× 10**^**3**^**/μl)**					
Min.–Max.	201.0–347.0	127.0–410.0	116.0–397.0	127.0–329.0	0.965
Mean ± SD.	253.9 ± 53.70	257.9 ± 101.5	245.1 ± 75.77	255.8 ± 67.10	
**WBCs (× 10**^**3**^**/μl)**					
Min.–Max.	4.0–8.38	3.48–8.93	4.25–14.30	3.48–7.83	0.856
Mean ± SD.	6.01 ± 1.42	6.42 ± 1.90	6.24 ± 2.42	5.80 ± 1.27	
**Neutrophils (× 10**^**3**^**/μl)**					
Min.–Max.	1.50–4.82	1.33–5.07	1.74–9.58	2.08–5.45	0.669
Median	3.03	2.96	3.12	3.77	
**Lymphocytes (x10**^**3**^**/ul)**					
Min.–Max.	1.46–2.72	0.96–3.40	0.87–4.05	0.80–2.40	0.022^*^
Median	1.92	2.42	2.03	1.66	
**ESR (mm/h)**					
Min.–Max.	8.00–35.00	7.00–60.00	5.00–70.00	10.00–72.00	0.038*
Mean ± SD.	20.75 ± 10.00	18.96 ± 18.33	29.57 ± 17.73	37.33 ± 21.11	
**CRP (mg/dl)**					
Min.–Max.	0.60–12.90	0.75–5.01	0.64–23.00	1.38–20.70	0.009*
Mean ± SD.	4.10 ± 3.90	2.91 ± 1.22	7.82 ± 6.63	9.91 ± 5.76	
**PDW (fl)**					
Min.–Max.	11.60–14.30	10.50–15.10	10.90–16.70	11.00–14.90	0.536
Mean ± SD.	12.98 ± 1.12	12.28 ± 1.37	13.10 ± 2.00	13.14 ± 1.19	
**MPV (fl)**					
Min.–Max.	7.70–11.80	7.10–13.50	7.60–12.20	0.20–13.50	0.651
Median	10.0	10.15	10.10	9.80	
**RDW (%)**					
Min.–Max.	12.40–16.80	12.40–18.0	11.10–18.0	10.20–16.90	0.579
Median	13.35	13.50	14.0	14.10	
**NLR**					
Min.–Max.	0.59–2.66	0.58–2.72	0.75–4.02	1.45–4.95	0.018*
Median	1.55	1.32	1.66	2.19	
**PLR**					
Min.–Max.	73.80–198.17	59.11–198.99	58.72–302.89	81.80–338.87	0.027*
Median	128.34	115.81	124.59	174.81	

*P* value ≤0.01: highly significant

*DAS* Disease activity score, *LDA* Low disease activity, *MDA* Moderate disease activity, *HDA* High disease activity, *RBCs* Red blood cells, *WBCs* White blood cells, *ESR* Erythrocyte sedimentation rate, *CRP* C reactive protein, *PDW* Platelet distribution width, *MPV* Mean platelet volume, *RDW* Red cell distribution width, *NLR* Neutrophil-lymphocyte ratio, *PLR* Platelet lymphocyte ratio

*Statistically significant at *P*≤ 0.05

Neutrophil-to-lymphocyte ratio, PLR, ESR, and CRP were significantly higher in patients with high disease activity using DAS-28 CRP (Table 3). Also, a statistically significant positive correlation was detected between NLR and PLR and both clinical disease activity scores (Table 4). RDW but not PDW was significantly higher in RA patients but both parameters had no association or correlation with clinical disease activity scores (Tables 1, 2, 3, and 4).

**Table 4:** Correlation between the hematological marker and different parameters

	Laboratory investigation									
	NLR		PLR		RDW		MPV		PDW	
	***r***_***s***_	***P***	***r***_***s***_	***P***	***r***_***s***_	***P***	***r***_***s***_	***P***	***r***_***s***_	***P***
**Disease duration (years)**	0.223	0.104	0.219	0.111	0.333*	0.014*	0.239	0.081	0.308*	0.024*
**TJC**	0.276*	0.043*	0.295*	0.031*	0.091	0.514	0.071	0.608	0.061	0.660
**SJC**	0.345*	0.011*	0.187	**0.176**	0.258	0.060	− 0.081	0.561	0.149	0.281
**VAS (mm)**	0.348*	0.010*	0.269*	0.050*	0.252	0.066	− 0.014	0.921	0.139	0.315
**ESR (mm/hr)**	0.072	0.605	0.295*	0.031*	0.042	0.763	0.189	0.171	− 0.061	0.661
**CRP (mg/dl)**	0.154	0.265	0.043	0.757	0.082	0.554	0.163	0.240	0.011	0.937
**DAS 28 (ESR)**	0.305*	0.025*	0.314*	0.021*	0.133	0.336	0.081	0.560	0.043	0.755
**DAS 28 (CRP)**	0.327*	0.016*	0.283*	0.038*	0.157	0.257	0.062	0.656	0.119	0.391

*r* Pearson coefficient, *r*_*s*_ Spearman coefficient

*TJC* tender joint count, *SJC* swollen joint count, *VAS* visual analog scale, *ESR* erythrocyte sedimentation rate, *CRP* C reactive protein, *DAS* disease activity score, *PDW* platelet distribution width, *MPV* mean platelet volume, *RDW* red cell distribution width, *NLR* neutrophil-lymphocyte ratio, *PLR* platelet lymphocyte ratio

*Statistically significant at *P*≤ 0.05, *P* value ≤0.01: highly significant

Both NLR and PLR had a statistically significant positive correlation with tender joint count (TJC), visual analog scale (VAS), and both clinical DAS 28 scores. In addition, NLR is positively correlated with swollen joint count (SJC). RDW and MPV are positively correlated with only disease duration. PDW was not correlated with any clinical or laboratory parameters of disease activity (Table 4).

**Fig. 1:**
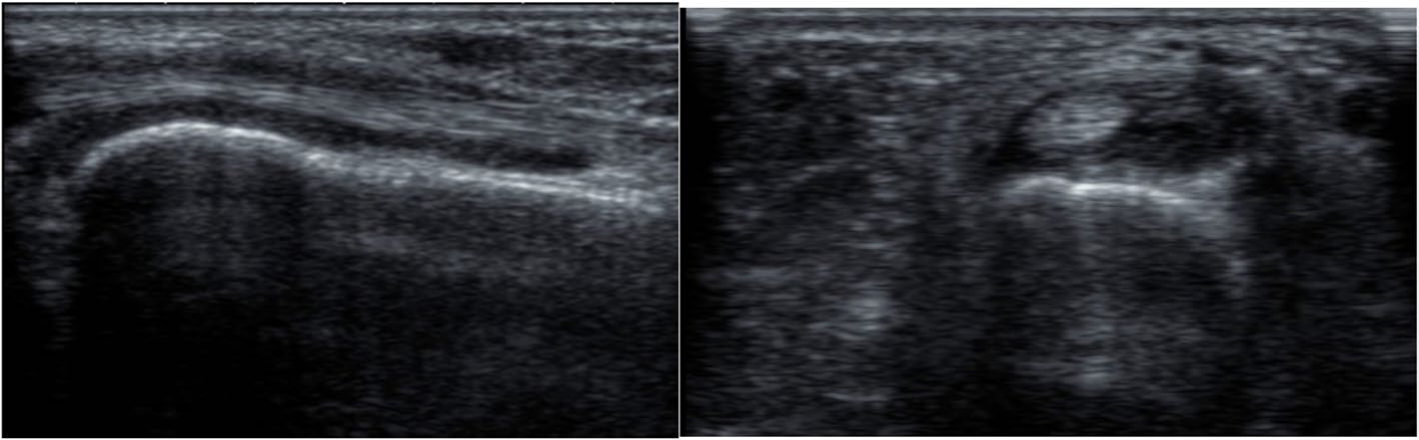
GSUS dorsal longitudinal and transverse scan of wrist joint showing grade II tenosynovitis

**Fig. 2:**
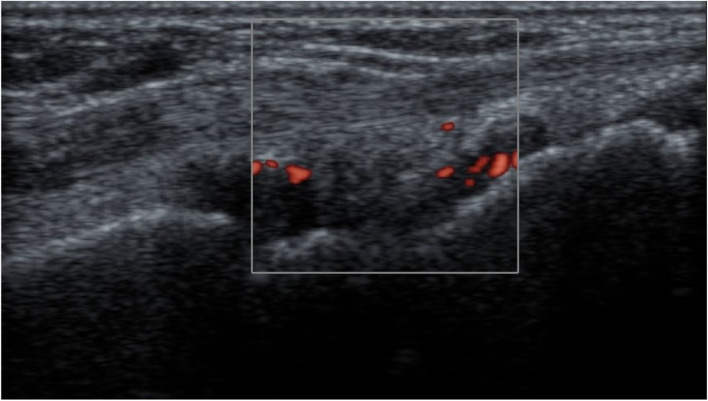
PDUS dorsal longitudinal scan showing grade II Doppler activity in wrist joint

**Fig. 3:**
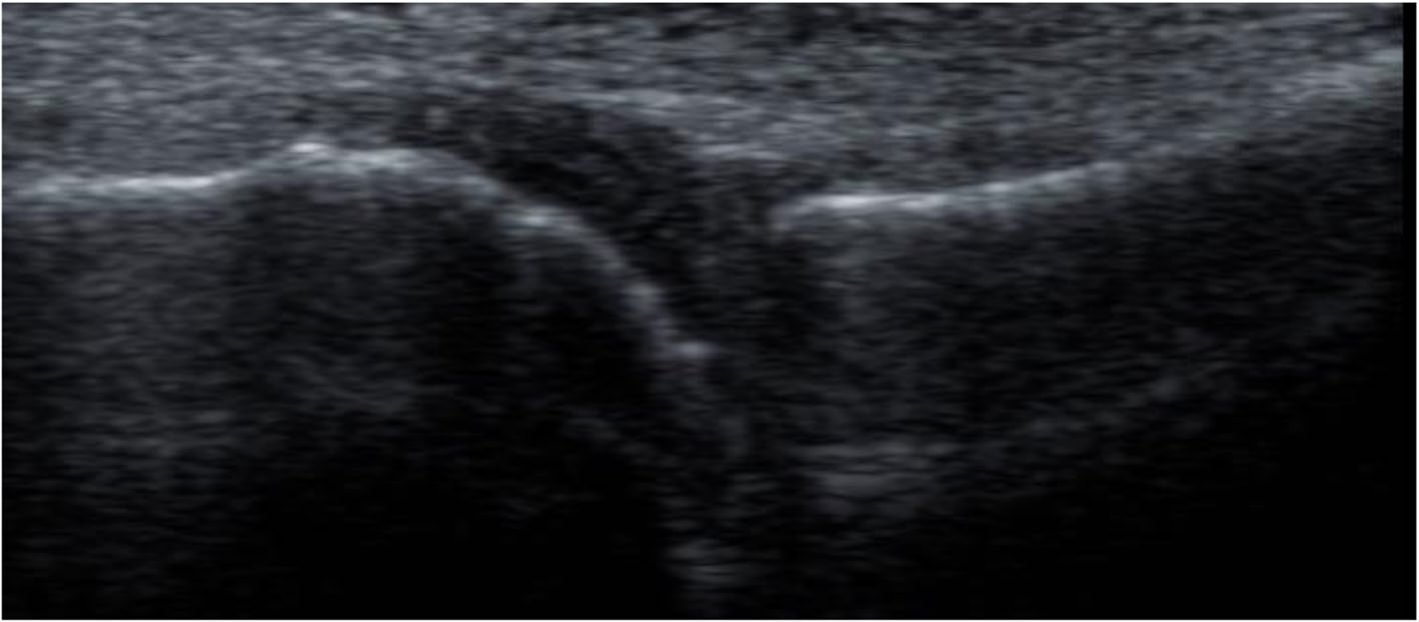
GSUS dorsal transverse scan showing grade II synovitis in the second MTP joint

According to the US7 score, the GS scores had a mean of 6.02 ±2.86 for synovitis and 2.85 ±1.61 for tenosynovitis/paratenonitis. The PD scores had a mean of 5.63 ±3.05 for synovitis and 3.22 ±2.29 for tenosynovitis/paratenonitis. The erosion score had a mean of 0.26 ±0.56 (Figs. 1, 2, and 3).

Ultrasonographic activity was scored in more than 92.6% of RA patients, while 7.4% showed ultrasonographic remission. After analysis of the clinical and ultrasonographic data, the US7 score was able to detect sonographic activity in 55.6% of patients classified as having remission using clinical DAS28-ESR score and in 62.5% using the DAS28-CRP.

NLR was found to have a statistically significant positive correlation with only tenosynovitis score using PDUS. ESR had a statistically significant positive correlation with GS synovitis score, PD synovitis, and tenosynovitis scores of US7 score. CRP was found to have a statistically significant positive correlation with only PDUS synovitis score. There were statistically significant positive correlations between DAS 28 ESR and CRP scores and synovitis and tenosynovitis scores by GS and PD ultrasound using the US7 score (Table 5).

**Table 5:** Correlation between US7 score and laboratory investigation and DAS

	US7 score									
	GSUS				PDUS				Erosion	
	Synovitis		Tenosynovitis		Synovitis		Tenosynovitis			
	***R***	***P***	***r***_***s***_	***P***	***r***_***s***_	***P***	***r***_***s***_	***P***	***r***_***s***_	***P***
**PDW (fl)**	0.022	0.872	− 0.007	0.961	− 0.052	0.707	0.043	0.757	− 0.053	0.706
**MPV (fl)**	− 0.029	0.834	0.014	0.917	0.102	0.462	0.034	0.809	0.037	0.788
**RDW (%)**	0.196	0.155	0.106	0.444	0.201	0.144	0.127	0.359	0.191	0.167
**NLR**	0.067	0.628	0.225	0.103	0.113	0.416	0.280^*^	0.041^*^	0.150	0.279
**PLR**	0.141	0.308	0.207	0.133	0.201	0.146	0.240	0.081	0.146	0.291
**ESR (mm/h)**	0.506^*^	<0.001^*^	0.295	0.031	0.626^*^	<0.001^*^	0.422^*^	0.001^*^	0.035	0.799
**CRP (mg/dl)**	0.214	0.120	0.137	0.322	0.271^*^	0.047^*^	0.176	0.204	0.082	0.555
**DAS28 ESR**	0.543^*^	<0.001^*^	0.390^*^	0.004^*^	0.523^*^	<0.001^*^	0.519	<0.001^*^	0.112	0.420
**DAS28 CRP**	0.453^*^	0.001^*^	0.365^*^	0.007^*^	0.421^*^	0.002^*^	0.470^*^	<0.001^*^	0.145	0.295

*r* Pearson coefficient, *r*_*s*_ Spearman coefficient

*PDW* Platelet distribution width, *MPV* Mean platelet volume, *RDW* Red cell distribution width, *NLR* Neutrophil lymphocyte ratio, *PLR* Platelet lymphocyte ratio, *ESR* Erythrocyte sedimentation rate, *CRP* C reactive protein, *DAS* Disease activity score, *GSUS* Grayscale ultrasound, *PDUS* Power Doppler ultrasound, *US* Ultrasound

*Statistically significant at *P*≤ 0.05, *P* value ≤0.01: highly significant

## Discussion

Complete blood count parameters, which are readily available in the clinical laboratory, can provide important information to the treating physician that helps in the effective management of patients with arthritis, according to Shiva et al. [33]. In this context, CBC was done for all patients enrolled in the study. Comparison between patients and controls showed a statistically significant difference between both groups regarding hemoglobin level and RBCs count being lower level in RA patients. In the literature, anemia is a common extra-articular manifestation of RA with an estimated prevalence ranging from 33.3 to 59.1%. Anemia of chronic disease is the most prevalent form followed by iron deficiency anemia which is comparable with our results [34–36]. Kumari et al. [37] reported a higher prevalence of anemia in RA patients (about 84%) which was higher than that found in our study (55.6%). This may be due to the difference in anemia definition criteria between study groups [37]. The decreased neutrophils count in our patients could be due to the effect of drugs used especially methotrexate which is the most commonly used synthetic DMARDs in RA treatment and has been proven to induce apoptosis of circulating neutrophils effectively [38].

As regards RDW, there was a statistically significant difference between the two groups as regards RDW, with no correlation with different disease activity assessment tools. Tecer et al. [39] found RA patients without anemia had higher RDW values when compared to healthy controls, and they concluded that RDW may be influenced by inflammation even in the absence of anemia. In contrast, this increased RDW may be mainly an effect of anemia rather than a true inflammatory index as mentioned by Lin et al. [40].

Regarding the platelets count in our study, there was no statistically significant difference between patients and control. This was in agreement with Uslu et al. [41], Zhang et al. [42], Boulos et al. [43], and Jin et al. [15] and is not in agreement with many researchers [5, 44–46]. This difference in results may be due to different sample sizes and study designs.

The MPV reflects the size of platelets in circulation and its elevation is suggestive of platelet production and activation in bone marrow, and it may be used as an indicator for the severity of inflammation [14, 39] while PDW reflects the range of variability in the platelet size and it has been evaluated as a marker of platelet morphology and activation [47]. It is considered a more specific indicator of platelet reactivity than the MPV because it remains unaffected by single platelet distention caused by platelet swelling [48].

In this study, there was no statistically significant difference between patients and control as regards MPV or PDW. Also, there was a statistically significant association between DAS 28 ESR and MPV only. No correlation was found between MPV or PDW with ESR or CRP.

In addition, MPV had a statistically significant positive correlation with hemoglobin level only. But, PDW had a statistically significant negative correlation with hemoglobin level and a positive correlation with disease duration. Conflicting data in the literature concerning the values of platelets, MPV, and PDW in patients with RA were found.

Khaled et al. [8] studied 98 RA patients and 53 matched controls and revealed a statistically significant difference between the two groups as regards PDW but not for MPV. Also, in our study, a statistically significant positive correlation was found between DAS28 and PDW and platelet count, and no correlation was found between it and MPV which was in accordance with Talukdar et al. [49]. But, Tacer et al. [39] who conducted their study on 154 RA patients showed that MPV levels were significantly higher in patients than control, but a significant association was not detected between MPV and DAS28.

The controversy among studies can be explained by the fact that regulation of platelet function and aging, is dependent on the maturity of thrombopoietic progenitors and different cytokines in the circulation, such as IL-6, IL-1, and TNF-α which affect platelet production and activation as they stimulate precursor cells of blood platelets. That results in a time-dependent change of platelet indices [13].

Moreover, the platelet indices have been shown to be sensitive to differences in blood sample anticoagulation, storage temperature, and delays in processing. Therefore, the lack of agreement may reflect differences in the assessed pathways of platelet activation as well as diurnal variation of MPV in both physiological and pathological conditions [8, 50, 51].

Neutrophils and lymphocytes have been reported to play an important role in the control of inflammation and are altered secondary to inflammation [15]. There was a lot of variation between studies when it came to the absolute neutrophilic and lymphocytic count in RA patients. In accordance with our results, Mercan et al. [2], Fawzy et al. [52], and Mohammed et al. [44] found a significant difference in absolute neutrophil count and a non-significant difference in lymphocytic count between patients and controls, while others showed a non-significant difference in both [5, 42, 45]. However, some studies showed a significant increase in both [15, 46, 53]. The decreased neutrophil count in our patients could be due to the effect of drugs used, especially methotrexate, which is the most commonly used synthetic DMARD in RA treatment and has been proven to induce apoptosis of circulating neutrophils effectively [38]. In addition, neutropenia may also be caused by deficiencies in folic acid, vitamin B12, or copper [54].

The NLR and PLR have drawn attention for many years as novel non-specific inflammatory markers [15]. Moreover, several reports have suggested the potential utility of both of them as diagnostic biomarkers in RA and other rheumatic diseases [5, 42, 55–57].

The NLR represents the two arms of the immune system: the innate system represented by the neutrophils and the adaptive system represented by the lymphocytes. This theoretical evidence explains the use of NLR in quantifying inflammation [58].

In this study, there were no statistically significant differences between patients and controls as regards NLR and PLR. However, both were significantly higher in patients with high disease activity using DAS-28 CRP. Also, a statistically significant positive correlation was detected between NLR and PLR with TJC, VAS, and both clinical DAS 28 scores. In addition, NLR is positively correlated with SJC.

Mohammed et al. [44] conducted their study on 50 RA patients and 20 control subjects and revealed a statistically significant difference between patients and controls as regards NLR and PLR, which was in disagreement with our results. NLR was correlated in their study with the DAS score but PLR had no correlation with disease activity scores.

Another meta-analysis by Gasparyan et al. [59] showed that combining PLR-NLR ROC curves can distinguish more accurately between patients with active RA and healthy controls and non-active RA patients from healthy controls.

Also, Jin et al. [15] conducted a multicenter retrospective study on 1009 RA patients, 170 patients with other rheumatic diseases, and 245 healthy controls from four medical centers and found a statistically significant difference between patients and controls as regards NLR and PLR, with their levels higher in RA patients than in other rheumatic diseases.

These discrepancies in results may be attributed to the small sample size of either patients or controls, as well as the presence of clinical heterogeneity. Moreover, variability in NLR has been reported in different races and is associated with personal and behavioral factors. In addition, the impact of specific immunosuppressive drugs on NLR and PLR could be one of the causes [58].

However, the statistically significant positive correlation between NLR or PLR and DAS28 CRP in our study group, as well as in other studies [58–60], suggests a possible relationship between inflammation and both markers.

One of the most important disadvantages of ESR and CRP is that, in spite of being good indicators of systemic inflammation, they may not reflect localized minimum disease activity [58]. It is notable that RA registry data for over 9000 patients has shown that more than half did not have elevations of ESR or CRP but had ongoing disease activity as determined by joint counts and global assessments [61]. Therefore, more sensitive and specific tools are required to assess or indicate the presence of inflammation in RA [58].

Our ultrasonographic examination tool was the US7 score, which has good feasibility and responsiveness according to OMERACT [62]. In addition, it has good inter-observer reliability and good performance when compared to the laboratory and clinical information [62, 63]. Moreover, if positive GS and PD signals are detected in this score, erosive changes and the occurrence of flares in patients in remission can be predicted [64].

Therefore, 92.6% of our cohort had ultrasonographic activity, whether synovitis was detected in GS or PD examination, while 7.4% showed ultrasonographic remission. For all patients, synovitis detected with GS and PD had the highest scores, ranging from 1 to 13 for GS and 0 to 11 for PD. Tenosynovitis and paratenonitis had lower scores, ranging from 0 to 5 for tenosynovitis detected by GS and 0 to 9 for PD signals in tenosynovitis. In addition, the erosion score ranged from 0 to 2. This was consistent with Meshwari et al. [19], who conducted a study on 61 RA patients with a comparable range in GS and PD scores. In addition, in agreement with our results, Targonska-Stepniak et al. [60] showed a positive correlation of both ESR and CRP and disease activity score with GS and PD ultrasonographic studies.

Higher scores were recorded by Kamel et al. [65]. This can be explained by the much higher percentage of patients with high disease activity in their study (58%) than in our study (only 27.8%).

The US7 score was able to detect subclinical activity in 55.6% of patients diagnosed as being in remission using the clinical DAS28-ESR score and 62.5% using DAS28-CRP. This was in agreement with El-Serougy et al. [66], who performed their study on 50 RA patients in remission according to DAS28, who found that 72% of their patients had ultrasonographic activity despite clinical remission. In addition, Amin et al. [67] conducted their study on 50 RA patients in clinical remission and LDA by DAS28-ESR and found that 61.8% of patients in clinical remission had subclinical synovitis detected by US.

A possible explanation for this finding may be that ESR and CRP are common inflammatory parameters and are good indicators of systemic inflammation, but they may not reflect localized, subclinical inflammation [60]. Therefore, the DAS 28 definition of remission has never been validated and may be insufficient because patients who achieve this cutoff point may still have high levels of inflammation and erosive damage [68].

A statistically significant positive correlation was found with only the tenosynovitis score by PDUS, but no correlations were found between components of the US7 score and PLR, MPV, or RDW. This was in disagreement with Targonska-Stepniak et al. [60], who had a statistically significant positive correlation between both NLR and PLR and GS and PD ultrasonography. This can be attributed to the small size of the study group.

## Limitations of the study

First, the study was at the same time of the COVID-19 pandemic. Second, the number of patients and control subjects was small and need to be increased for more reliable results about the change of the studied inflammatory markers according to disease activity. Third, we did not monitor the effect of drugs on different inflammatory markers and the effect of patient treatment compliance.

## Conclusions

Neutrophil-to-lymphocyte ratio, platelet-to-lymphocyte ratio, and mean platelet volume could be potential inflammatory markers for follow-up of disease activity in RA activity. The ultrasound 7 score is a simple and practical scoring system for use in the detection of inflammation, even subclinically in RA patients, which may help the physician in his clinical decisions. The combined use of both hematological markers and the ultrasound 7 score may be of great value. Well-designed, prospective cohort studies with a larger sample size are needed to prove the clinical value, sensitivity, and specificity of different hematological markers as inflammatory markers in RA patients, either alone or in combination with the ultrasound 7 score.

## Data Availability

Available.

## Abbreviations

RA: Rheumatoid arthritis
CRP: C-reactive protein
ESR: Erythrocyte sedimentation rate
NLR: Neutrophil-to-lymphocyte ratio
PLR: Platelet-to-lymphocyte ratio
PDW: Platelet distribution width
RDW: Red cell distribution width
MPV: Mean platelet volume
US: Ultrasound
DAS: Disease activity score
CBC: Complete blood count
RBCs: Red blood cells
WBCs: White blood cells
GS: The Gray Scale
PD: Power Doppler
LDA: Low disease activity
MDA: Moderate disease activity
HAD: High disease activity
MCP: Metacarpophalangeal joint
TJC: Tender joint count
SJC: Swollen joint count
VAS: Visual analog scale
PIP: Proximal interphalangeal joint
MTP: Metatarsophalangeal joint
DAS: Disease activity score
